# Morphology Regulation of Zeolite MWW via Classical/Nonclassical Crystallization Pathways

**DOI:** 10.3390/molecules29010170

**Published:** 2023-12-27

**Authors:** Wenwen Zi, Zejing Hu, Xiangyu Jiang, Junjun Zhang, Chengzhi Guo, Konggang Qu, Shuo Tao, Dengran Tan, Fangling Liu

**Affiliations:** 1 School of Chemistry and Chemical Engineering, Liaocheng University, Liaocheng 252000, Chinaqukonggang@lcu.edu.cn (K.Q.); taoshuo@lcu.edu.cn (S.T.);; 2 State Key Laboratory of High-Efficiency Utilization of Coal and Green Chemical Engineering, College of Chemistry and Chemical Engineering, Ningxia University, Yinchuan 750021, China; zhangjj089@nxu.edu.cn

**Keywords:** zeolite, synthesis, morphology, crystallization mechanism

## Abstract

The morphology and porosity of zeolites have an important effect on adsorption and catalytic performance. In the work, simple inorganic salts, i.e., Na salts were used to synthesize MWW zeolite using the organic compound 1-Butyl-2,3-dimethyl-1H-imidazol-3-ium hydroxide as a structure-directing agent and the morphology was regulated by the alkali metals. The sample synthesized without Na salts shows a dense hexagon morphology, while different morphologies like ellipsoid, wool ball, and uniform hexagon appear when using NaOH, Na_2_CO_3_, and NaHCO_3_, respectively. Moreover, the impact of Na salts on the induction, nucleation, and the evolution of crystal growth was studied. Different kinds of Na salts have a different impact on the crystalline induction time in the order of NaHCO_3_ (36 h) < Na_2_CO_3_ (72 h) = NaOH (72 h). Meanwhile, the crystalline mechanism with the cooperation of inorganic salts and the organic SDAs is proposed. NaOH- and Na_2_CO_3_-MWW zeolite crystallized with a network of hydrogel via the nonclassical pathway in the system; however, the product is synthesized via a classical route in the NaHCO_3_ environment. This work provides information about MWW zeolite crystallization and modulating diverse morphologies by adjusting the process.

## 1. Introduction

MWW zeolite consists of 10 membered rings (MRs) (4 Å × 5.9 Å), a two-dimensional interlayer sinusoidal channel and the 12-ring supercages (7.1 Å × 7.1 Å × 18.2 Å) connected by 10 MR apertures, which have been widely investigated acting as efficient catalysts in the alkylation, isomerization, and disproportionation reactions [1,2,3,4]. The zeolite type includes MCM-22 [5], MCM-36 [6], MCM-49 [7], MCM-56 [8], ITQ-1 [9], ITQ-2 [10], SSZ-25 [11], etc. The morphology and porosity of MWW zeolite is closely related to the utilization efficiency of catalytic activity [12,13,14,15]. The sole microporous system not only leads to catalyst deactivation and consequently unfavorable catalyst regeneration but also restricts their applications in the bulky-molecule-involved process. Therefore, introducing mesopores with the size of 2–50 nm is a good choice, which can largely increase the accessibility of active sites in the zeolite framework [16,17]. The templates such as the surfactants, gels, porous solids, and some designed structure-directing agents can have an impact on the crystal engineering [18,19]. However, the pollutant of the surfactants limits the viability of the technique. Therefore, modulating the zeolite morphology by the employment of soft or dual-soft templates and directing control of the synthesis conditions, such as molar composition, pH, and temperature is more preferred [20,21]. These can influence the kinetics and thermodynamics of crystal growth, which is not only executable but also environmentally friendly, and thus build complex multilevel structures from a functional basic unit.

**Scheme 1:**
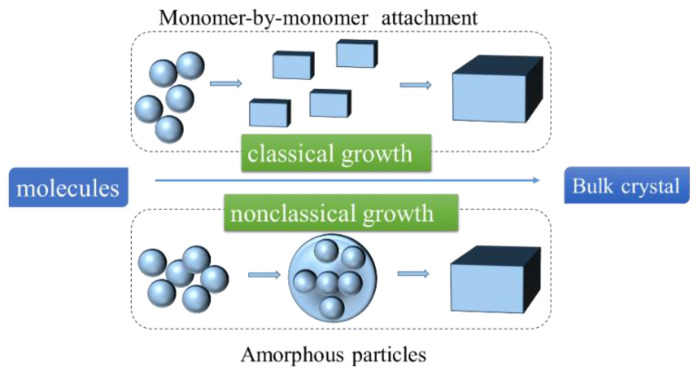
Two pathways of zeolite crystallization.

Understanding the zeolite crystalline mechanism is of significance for adjusting its morphology more scientifically [22,23,24]. As far as we know, investigation of the MWW zeolite crystallization mechanism is rare. Generally speaking, zeolite can be formed by the classical (i.e., monomer addition) and nonclassical (i.e., CPA, particle attachment) mechanisms, and it remains controversial because of the complicated synthesis systems (Scheme 1) [25,26,27]. The former holds the view that the zeolites are formed from the simple species like atoms or molecules to a preformed nucleus, while the latter involves the attachment of precursors ranging from oligomers to amorphous particles and small crystallites [28,29]. The nucleation and crystallization rates, which are used to describe the zeolite crystallization process, depend on the crystallization chemistry of the system, like the state of the precursors and the intermediate species [30,31]. There are studies which state that varying the supersaturation of the system solution can switch the pathway from the nonclassical pathway to the classical one, further affecting the morphology of the product [32,33]. The effect of alkali metals ion on the formation of structure and morphology was discussed and the crystallization process was re-examined, which emphasizes the role of the alkali metal ions [12,22,34].

Herein, we synthesized a microporous MCM-22 zeolite with sheetlike building block morphology by using the cheap imidazolium and Na salts. Of note, diverse morphologies with relatively loose structure different from the initial dense microcrystalline structure appeared after cheap inorganic salts were added into the system, which suggests the simple method for tailoring the sample morphology is effective. The crystallization processes of the products and the possible crystallization mechanism have been explored. Our findings reveal the effect of simple inorganic salts on the morphology and porosity as well as the formation process without introducing other surfactants, providing directions for regulating zeolite morphology and the crystallization pathways.

## 2. Results and Discussion

### 2.1. Synthesis of MWW Zeolites Only with the Organic Structure-Directing Agent

**Figure 1:**
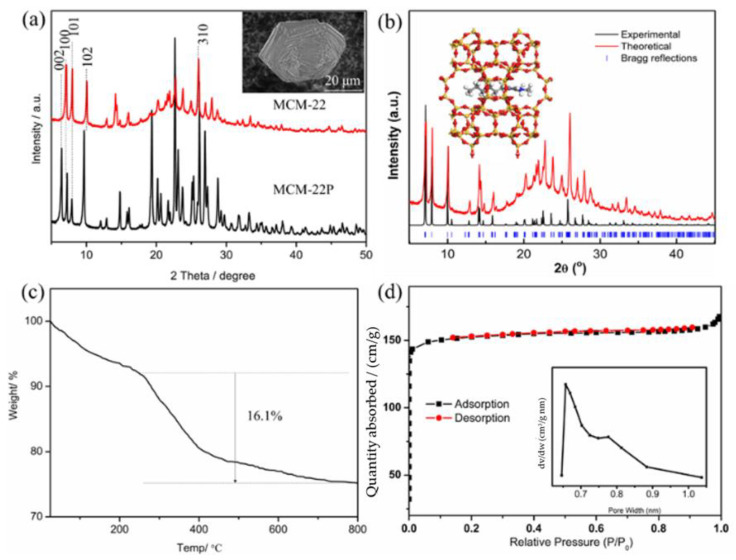
(**a**) PXRD patterns of the synthesized MCM-22P and the calcined sample. (**b**) Rietveld refinements of MCM-22 and the structure with SDA inside. (**c**) TG thermogravimetry of the pure silica MWW zeolite synthesized with the only SDA. Experimental (red) and calculated (black) PXRD patterns as well as the positions of the Bragg reflections shown by the short tick marks (blue). (**d**) N_2_ isotherm curve of the calcined pure silicate MCM-22.

MCM-22P (the precursor of MCM-22 zeolite) can be synthesized using 1-Hexyl-2,3-dimethyl-1H-imidazol-3-ium hydroxide as the only structure-directing agent. The four peaks at 6.4°, 7.2°, 7.9°, and 9.7° (Figure 1a) corresponding to (002), (100), (101), and (102), respectively, appear as the characteristic of the MCM-22P. The first two peaks become one at 7.3° and the intralayer (002) reflection overlaps with the (100) after being calcined under air atmosphere due to the condensation of the layer. The sample exhibits a large micrometer scale morphology with about 40 μm as seen from the inset. According to the structure mechanic simulations, the organic structure-directing agent was located inside the framework structure (Figure 1b inset). The PXRD pattern is well fitting the simulated one. TG curve (Figure 1c) shows a loss of about 16.1% due to the decomposition of SDA, suggesting the amount of the organic species occluded in the micropores. The obtained MWW zeolite showed type I sorption isotherms (Figure 1d) with a large BET surface area of 620 m^2^/g with a pore size distribution at 0.68 Å and a little pore volume of 0.26 cm^3^/g, indicating a typical microporous structure. According to the solid-state ^13^C NMR spectra, peaks of the imidazolium cations in as-synthesized MCM-22P samples matched well with those dissolved in the solvent (Figure S1), indicating the SDA cations in the solid sample were intact.

### 2.2. The Effect of Na Salts on the Hierarchical Structure

**Figure 2:**
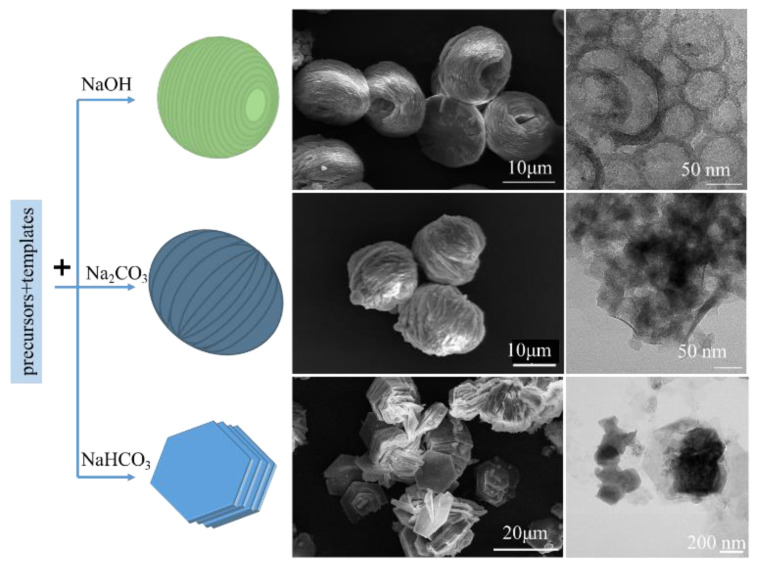
Paragraphs including the SEM, N_2_ isotherm curves, and TEM images of MCM-22P using SDA directly and using the cooperation of SDA with NaOH, Na_2_CO_3_, and NaHCO_3_ under the same ratio of Na^+^/Si = 0.1.

Previously, a high-order doughnutlike structure assembled by zeolitic building blocks was in situ synthesized using a supramolecular surfactant agent C_16_H_33_(OC_2_H_4_)_20_OH [35]. In the study, DTA (differential thermal analysis) investigations were undertaken to determine the role of nonionic surfactant. It was supposed that the interaction of repeating oxyethylene units with the aluminosilicates led to the assembly of the superstructures. However, in the work, we found that a similar morphology can be produced by the simple Na salts. The pure silica sample changes from a dense structure to a loose one with the addition of Na salts (NaOH, Na_2_CO_3_, NaHCO_3_). PXRD patterns of all the products in Figure S2 show the pure MCM-22P phase with high crystalline, indicating the addition of Na salts does not cause any impurity peaks. As seen from Figure 2, when NaOH was added, the sample shows an ellipsoid morphology with a shallow hole in the center, Na_2_CO_3_ with a wool shape like many lines circling the sphere and NaHCO_3_ displayed a hexahedron morphology in the form of stacked ones or a single one. Although alkali salts were used to synthesize MTW, MFI, and MWW and so on, little research about MWW zeolite mentioned the role of Na salts [13,15,22,36,37]. Moreover, different morphologies are related to the used organic structure-directing agent. Our obtained results are supposed to be related to the effect of the cooperation of Na salts and the used organic directing agent (1-Hexyl-2,3-dimethyl-1H-imidazol-3-ium hydroxide) and their interaction with the silcates.

To verify the effect of NaOH on the zeolite, the amount of NaOH was adjusted from 0.05 to 0.25 relative the molar of Si under a certain amount of HF and SDAs. To verify the products are silica-based materials, EDS results are shown in Table S1. As shown in Figure S3, with low NaOH, some nanoparticles can be seen, although big micron-level sphere morphology has appeared. Increasing the amount of NaOH, more regular morphologies with the thicker edge, and the thinner circle could be observed, and the middle shallow circle becomes more apparent. The feature of the morphology can be seen more clearly from the TEM image (Figure 2), showing that the sample has a hierarchical structure with voids, which makes a supplement for the SEM results. N_2_ adsorption–desorption isotherms of sorption of the related materials show type IV isotherms with an H4 hysteresis loop in Figure S4. The BET result changes with increasing the amount of NaOH and all samples display a low external surface area compared with the pure silica MWW zeolite without the addition of Na (Table 1). In all the pore distribution curves, no obvious mesopore is detected in NaOH-MWW except a pseudopeak at 3.8 nm. The results indicate the addition of NaOH alters the morphology although changing the amount of NaOH has no obvious effect on the zeolite structure, which verified the role of NaOH in the formation of the ellipsoid morphology structure.

**Figure 3:**
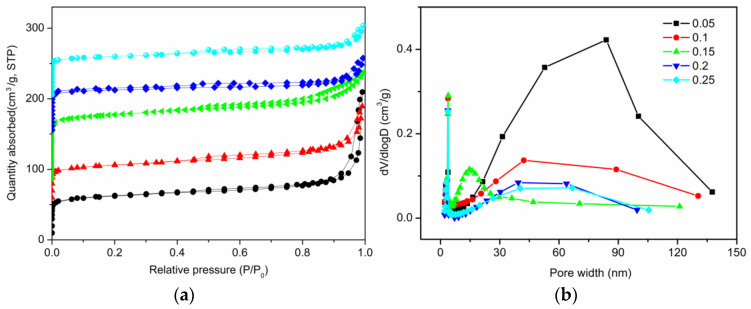
(**a**) N_2_ adsorption/desorption isotherms and (**b**) pore size distribution of the samples with the addition of NaHCO_3_ under different ratios of HF/Si (a: 0.5, b: 0.65, c: 0.75, d: 1, e: 1.25).

Under a certain amount of HF and SDAs, increasing the amount of Na_2_CO_3_ from 0.05 to 0.2 relative to the amount of Si, all the samples exhibit the wool ball morphology with a size of about 15 μm, as seen in Figure S5 and the EDS results are shown in Table S2. The typical SEM and TEM images of Na_2_CO_3_-added samples are displayed in Figure 2, which suggest the feature of the morphology. The N_2_ adsorption-desorption isotherms of the samples with different Na_2_CO_3_ have been tested as plotted in Figure S6 and the data are listed in Table 2. According to the result of the N_2_ isotherm data, it was supposed that a certain amount of Na_2_CO_3_ may destroy the zeolite structure. The integration of SEM images and N_2_ data indicates that the good-looking morphology is not so closely related to the microporous property. The sample with the ratio of Na_2_CO_3_/Si = 0.05 displays a normal N_2_ isotherm curve with a BET surface area of 218 m^2^/g and a total volume of 0.12 cm^3^/g. The results verify the feature of typical microporous materials, which is the same as that of NaOH. For the addition of NaHCO_3_, different morphology from the NaOH-MWW and Na_2_CO_3_-MWW was obtained. After increasing the amount of NaHCO_3_, the morphology shows no obvious change and no stable microporous materials were obtained. The EDS results are shown in Table S3 to suggest the samples are silica-based materials, rather than salts such as SiFx. But an interesting phenomenon can be found after varying HF with a certain amount of NaHCO_3_: the morphology changes from the stacked sphere to hexahedron with tunable mesopores while keeping the micropores (Figure S7), which suggests the HF content in the NaHCO_3_-added synthesis gel affects the zeolite particle morphology and the similar results do not show in the system without Na salts when varying the HF. Figure 2 shows the SEM and TEM image of the NaHCO_3_-MWW sample, in which the intracrystalline mesopore can be seen. The N_2_ adsorption-desorption isotherms of the samples with different NaHCO_3_ have been tested as plotted in Figure 3. Almost all the NaHCO_3_-MWW materials contain the intraparticle mesopores. The data for V_meso_ and S_ext_ (Table 3) decrease from 0.25 to 0.08 cm^3^ g^−1^ and from 61 to 39 m^2^ g^−1^ as the HF/SiO_2_ ratio changes from 0.5 to 1.25, respectively, which confirm the above result. The curve about the trendency was plotted as in Figure S8, which indicates the mesopore volume decreases when increasing the acidity of the system. Besides, TG analysis (Figure S9) was tested to account for the weight loss of the samples with different HF. When increasing the amount of HF, the weight loss displays the tendency of rising at first and then declining, suggesting the effect of the acidity of the system on the stability of the material, thereby affecting the porosity of the structure.

Based on the above study results, NaOH, Na_2_CO_3_, and NaHCO_3_ have different effects on the morphology and porosity of MWW zeolite. For the two former Na salts, it displays no obvious change with varying the solution concentration. For NaHCO_3_, as tuning the acidity of the system, the morphology and porosity change obviously, further suggesting the addition of NaHCO_3_ has a great impact on the structure.

### 2.3. Investigation on MWW Zeolite Crystallization

**Figure 4:**
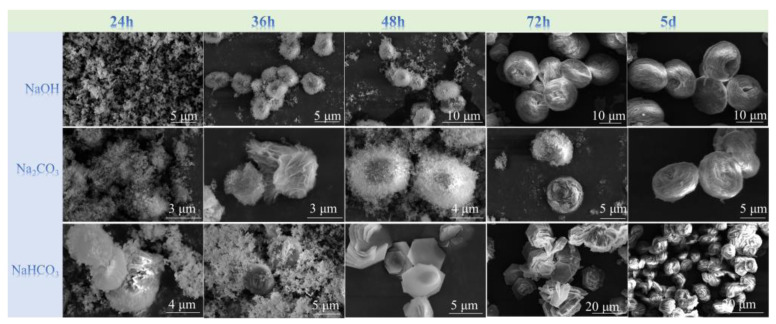
SEM pictures of the samples synthesized with Na salts at different crystalline time.

To investigate the evolution process of Na-MWW, the solid products from the synthesis mixture at different crystallization times from 24 h to 120 h were characterized by XRD and SEM. For NaOH-MWW samples, precursor nanoparticles were observed when the sample crystallized for 24 h, and the crystallization occurs by nanoparticle-by-nanoparticle attachment with the crystallization to 36 h as shown in SEM (Figure 4). From the SEM picture of 48 h, it can be seen that many small nanoparticles were still around the large aggregated ones, suggesting the state of attachment. When the reaction reached 72 h, the amorphous components were nearly completely consumed, and then gradually grew into large crystals. After 5 days, the sphere grew larger due to the Ostwald ripening and the crystallinity reached its highest. The change of the PXRD patterns of the samples is not so obvious until the 5th day when the integrated peaks are seen (Figure S10a).

For Na_2_CO_3_ added sample, on the first day, an amorphous haze was obtained judging from a broad reflection from 5 to 50°. After 48 h, weak PXRD peaks appear, the precursor aggregates with a particle size of about ca. 6 μm, as shown in SEM (Figure 4). The crystallinity of the sample markedly increased with the larger particle size between 48 and 72 h. PXRD patterns show the variation of the peaks (Figure S10b). The SEM images show the aggregated plate sphere particles with a size of about 5–15 μm. More crystals with high crystallinity were produced when the growth time increased to 5 d. These crystals synthesized with the addition of Na_2_CO_3_ were formed via the hydrogel by aggregates from the initial amorphous particles, which was the same as that of NaOH-MWW sample.

For the NaHCO_3_ added sample, the aggregated plates composed of nanoparticles appear at the first 24 h and the plates became thicker as time went by and were surrounded by the amorphous particles. Different from the round sphere above, hexahedron morphology was obtained at 48 h, and spiral growth can be observed, which displays the dissolution and crystallization process. From the PXRD patterns (Figure S10c), whole peaks with weak intensity were seen. With a prolonged reaction time of 72 h, single hexahedron grew into multilayers. In the crystallization process, hydrogel wasn’t seen, which suggested a different growth mechanism, i.e., nonclassical pathway.

For all the samples, crystallization almost finished accompanied by the emergence of (100), (002), (101), and (102) reflection planes with high peak intensity that appear on the third day. The small alkali cations have an effect on producing structures with different morphology, probably due to the different anions, which increased the alkalinity of the system. Although NaHCO_3_ turns into Na_2_CO_3_ after the reaction, different results from Na_2_CO_3_ were found maybe due to the chemical reaction step (HCO_3_^−^ + OH^−^= CO_3_^2−^ + H_2_O). Moreover, the salting-out effect of alkali metal cations can have an impact on the manner of aggregation or attachment, thereby the crystalline takes place by two different growth processes [22]. As a result, different nucleation, morphologies, and induction time in the order of NaHCO_3_ (36 h) < Na_2_CO_3_ (72 h) = NaOH (72 h) were obtained depending on the kinds of anions. The comparison of the induction time of the three kinds of Na salts was clearly shown in Figure S10d.

### 2.4. The Proposed Mechanism of Crystallization of MWW Zeolite

In the process of crystallization, three particle populations are observed: (1) precursor particles with a size of about ca. 100 nm; (2) aggregated primary units related to crystalline MWW structure; (3) micrometer sized MWW crystals.

**Scheme 2:**
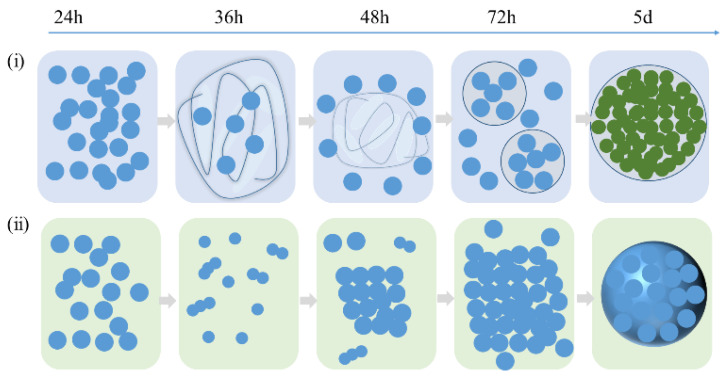
Crystallization pathways of MWW zeolite: NaOH/Na_2_CO_3_-added (**i**) and NaHCO_3_-added (**ii**).

Based on the above evolution process, a possible crystallization mechanism of the Na-induced MWW zeolite was proposed. Seeing from the SEM images, different crystallization mechanisms appear due to the addition of Na salts. The crystallization of NaOH/Na_2_CO_3_-MWW samples follows the aggregation–crystallization process (Scheme 2i) as follows: (1) in the first 24 h, the precursor particles gradually aggregated into larger particles; (2) the larger particles were further accumulated to a hydrogel network with the prolonged reaction time from 24 h to 36 h; (3) the network quickly changed into an ellipsoid shape with a micro size and low crystallinity when reaction prolonged to 72 h; (4) finally, a fine crystalline mesocrystal MWW zeolite with a rough surface is obtained. The above process was defined as the nonclassical crystallization mechanism. The NaHCO_3_-MWW sample crystallized by the following pathway (Scheme 2ii): (1) the nanoparticles began accumulating at the reaction of 24 h; (2) the larger aggregated precipitates further grew at 36 h instead of forming a hydrogel network; (3) a dense zeolite crystal with micro size and relatively smooth surface was formed via the spiral growth from 48 to 72 h; (4) a high crystalline structure with more regular morphology could be collected at the final stage of the crystallization. The crystalline process involves the particle attachment and layer-by-layer growth. It is therefore regarded as classical crystalline growth.

Generally, the crystallization of zeolite undergoes the following process: (1) first, amorphous nanoparticles gradually accumulate and aggregate; (2) then, nanoparticles grow through attachment or layer-by-layer and transform into different morphologies due to the different effect of Na salts; (3) bulk crystallized zeolite with regular and smooth surface morphology is obtained as the crystallization time extends. Above all, it is concluded that MWW zeolite is crystallized via a dynamic process from disordered species to the ordered ones, including the classical and nonclassical pathway, which provides some guidance for controlling the growth and shows promise for the excellent performance in various fields.

## 3. Materials and Methods

### 3.1. Materials

1-Hexyl-2,3-dimethylimidazolium chloride (98 wt%, Bide Pharmatech Co., Ltd., Shanghai, China), tetraethylorthosilicate (TEOS, 98%, Sinopharm, Shanghai, China), 717 anion exchange resin (Sinopharm), NaOH, Na_2_CO_3_, NaHCO_3_ (Sinopharm), hydrochloric acid (HCl, 36–38%, Sinopharm), and hydrofluoric acid (HF, 40%, Sinopharm) were directly used as purchased without further purification.

### 3.2. Preparation of MCM-22 Zeolite

The sample was synthesized using 1-Hexyl-2,3-dimethyl-1H-imidazol-3-ium hydroxide as the structure-directing agent (SDA) under the fluoride environment. The hydroxide forms of the SDA were obtained from the chloride by anion exchange. The concentration of the SDA(OH) was obtained by titration using 0.1 M HCl.

The products were prepared according to the refs. [38,39]. In a typical procedure, 1.5 mmol (335 μL) of TEOS was added into the SDA(OH) (4.5 mL, 0.75 mmol) solution under stirring for about an hour. Then an amount of Na salt was added for another 30 min, followed by the addition of HF (0.75 mmol, 33 μL, 40 wt%) under vigorous stirring. Next, the mixture was heated at 80 °C to remove excess water and hydrolyzed alcohol until the desired molar ratio of 1.0 SiO_2_: 0.75 SDA(OH): xNaOH/Na_2_CO_3_: 0.75 HF: 5/10 H_2_O was achieved, where x = 0, 0.05, 0.1, 0.15, 0.2, 0.25. The gel was crystallized in a 15-mL-Teflon-lined autoclave at 160 °C for 5 d. The products were collected by filtration, washing, and drying in air at 80 °C to evaporate excess water and ethanol until the desired water ratio was achieved. The product was recovered via filtration and washed with water and ethanol three times, and dried in air. The pristine sample was calcined at 550 °C under air for about 5 h to remove the SDA to obtain the final product.

For the sample synthesized with the addition of NaHCO_3_, the process is the same as the above except that the amount of NaHCO_3_/SiO_2_ was fixed as 0.1 while changing the amount of HF/SiO_2_ as 0.5, 0.65, 0.75, 1, and 1.25. To verify the effect of Na without introducing other heteroatoms and exclude the other impacts as much as possible, the simple three sources (NaOH, Na_2_CO_3_, and NaHCO_3_) were used. Meanwhile, the pure zeolite phase must be chosen in order to verify the effect of different Na salts; the amount of NaOH and Na_2_CO_3_ at a certain of HF and the effect of HF on NaHCO_3_ are thus investigated.

### 3.3. Characterization of Materials

Powder X-ray diffraction (PXRD) patterns were performed on a Rigaku Smartlab diffractometer using Cu Ka radiation (λ = 1.5418 Å) in the 2θ ranging from 5 to 50°. Scanning electron microscopy (SEM) images were obtained on a field emission scanning electron microanalyzer (Hitachi S-4800). The element content was measured by an Energy Dispersive Spectroscopy (EDS, Bruker). Transmission electron microscopy (TEM) was tested using a Thermo Fisher Talos electron microscope operating at 200 kV. Thermogravimetric analysis (TGA) was performed on a NETZSCH STA 449 F3 equipment. BET surface areas and the related data about the aperture were tested on a Micromeritics ASAP 2460 analyzer under 77 K. Solid-state ^13^C MAS NMR spectra were carried out on a Bruker Av-400 spectrometer using magic-angle spinning (MAS) techniques at 100.62 MHz.

## 4. Conclusions

Pure silica MWW zeolite can be synthesized using imidazolium as the structure-directing agent and dense morphology can be obtained. After adding different kinds of Na salts, the screwball, wool ball, and hexahedron morphologies were seen. With different kinds of Na salts, the induction crystallization time gradually increased in the order of NaHCO_3_ < Na_2_CO_3_ = NaOH. For Na_2_CO_3_ and NaOH addition, no obvious change was found in the pore system and the micropore domains. For NaHCO_3_ addition, varying the amount of HF brings about different porosity in addition to the micropore. The synthesis mechanism synthesized with Na salts was put forward, including the formed hydrogel via monomer addition and the classical mechanism from the amorphous precursor. The work provides some guidance for the synthesis of MWW zeolite with different morphology and porosity and opens perspective for controlling the acid site distribution in MWW and further catalysis application.

## Figures and Tables

**Table 1:** Texture properties of the calcined MWW zeolite with the addition of different ratio of NaOH/Si (H_2_O/Si = 5, HF/Si = 0.75).

Run	NaOH/Si	Surface Area (m^2^/g)			Pore Volume (cm^3^/g)		
		S_BET_^[a]^	S_micro_^[b]^	S_ext_^[c]^	V_total_	V_micro_^[d]^	V_mes_
1	0.05	208	170	38	0.12	0.07	0.05
2	0.1	228	194	34	0.13	0.08	0.05
3	0.15	167	146	21	0.09	0.06	0.03
4	0.2	191	175	16	0.11	0.07	0.04
5	0.25	214	190	24	0.11	0.08	0.03

^[a]^ BET surface area, ^[b]^ t-plot micropore surface area, ^[c]^ t-plot external surface area, ^[d]^ t-plot micropore volume.

**Table 2:** Texture properties of the calcined MWW zeolite with the addition of different ratio of Na_2_CO_3_/Si (H_2_O/Si = 5, HF/Si = 0.75).

Run	Na_2_CO_3_/Si	Surface Area (m^2^/g)			Pore Volume (cm^3^/g)		
		S_BET_^[a]^	S_micro_^[b]^	S_ext_^[c]^	V_total_	V_micro_^[d]^	V_mes_
1	0.05	218	177	41	0.12	0.07	0.05
2	0.1	38	24	14	0.03	0.01	0.02
3	0.15	118	102	16	0.07	0.04	0.03
4	0.2	126	97	29	0.08	0.04	0.04
5	0.25	26	7	19	0.05	0.003	0.047

^[a]^ BET surface area, ^[b]^ t-plot micropore surface area, ^[c]^ t-plot external surface area, ^[d]^ t-plot micropore volume.

**Table 3:** Texture properties of the calcined MWW zeolite with the addition of NaHCO_3_ under different ratio of HF/Si (H_2_O/Si = 10, NaHCO_3_/Si = 0.1).

Run	HF/Si	Surface Area (m^2^/g)			Pore Volume (cm^3^/g)		
		S_BET_^[a]^	S_micro_^[b]^	S_ext_^[c]^	V_total_	V_micro_^[d]^	V_mes_
1	0.5	229	168	61	0.32	0.07	0.25
2	0.65	211	148	63	0.22	0.06	0.16
3	0.75	375	297	77	0.24	0.12	0.12
4	1	240	211	29	0.17	0.08	0.09
5	1.25	229	190	39	0.16	0.08	0.08

^[a]^ BET surface area, ^[b]^ t-plot micropore surface area, ^[c]^ t-plot external surface area, ^[d]^ t-plot micropore volume.

## Data Availability

The data presented in this study are available upon request from the corresponding author.

## Supplementary Materials

The following supporting information can be downloaded at: https://www.mdpi.com/article/10.3390/molecules29010170/s1, Figure S1. Comparison of ^13^C NMR spectra of the synthesized sample and the liquid NMR in D_2_O. Figure S2. PXRD patterns of the samples with NaOH, Na_2_CO_3_, NaHCO_3_. Figure S3. SEM images of the samples with different ratio of NaOH/Si (a: 0.05, b: 0.1, c: 0.15, d: 0.2, e: 0.25). Figure S4. N_2_ adsorption/desorption isotherms and pore size distribution of NaOH/Si. Figure S5. SEM images of the samples with different ratio of Na_2_CO_3_/Si (a: 0.05, b: 0.25, c: 0.1, d: 0.15, e: 0.2). Figure S6. N_2_ adsorption/desorption isotherms and pore size distribution of Na_2_CO_3_/Si. Figure S7. SEM images of the samples with the addition of NaHCO_3_ under different ratio of HF/Si (a: 0.5, b: 0.65, c: 0.75, d: 1, e: 1.25) Figure S8. The effect of HF on the Sext and Vmes in the NaHCO_3_-MWW samples. Figure S9. (a) TG analysis of the samples with the addition of NaHCO_3_ under different ratio of HF/Si (a: 0.5, b: 0.65, c: 0.75, d: 1, e: 1.25) and (b) the mass loss comparation. Figure S10. PXRD patterns of samples with different kinds of Na salts subjected to the crystallization time and the comparison of the nucleation time. Table S1. EDS data for the samples with the addition of different ratio of NaOH/Si. Table S2. EDS data for the samples with the addition of different ratio of Na_2_CO_3_/Si. Table S3. EDS data for the samples with the addition of NaHCO_3_ under different ratio of HF/Si.

## References

[1] Wang T., Huang W., Han H., Zhang J., Wu H., Yan X., Jiang Y., Fang L., Zhang B., Guo X. (2022). Facile and fast synthesis of highly active Lewis acid MWW zeolite from pure silica ITQ-1. Inorg. Chem. Front..

[2] Bao R., Shen Z., Ma C., Li L., Wang Y., Sun H., Yang W. (2022). Delaminated MWW-type zeolite and its catalytic performance in alkylation of benzene with cyclohexene. Appl. Catal. A-Gen..

[3] Barakov R., Shcherban N., Petrov O., Lang J., Shamzhy M., Opanasenko M., Čejka J. (2022). MWW-type zeolite nanostructures for a one-pot three-component Prins–Friedel–Crafts reaction. Inorg. Chem. Front..

[4] Zhou Y., Mu Y., Hsieh M.-F., Kabius B., Pacheco C., Bator C., Rioux R.M., Rimer J.D. (2020). Enhanced Surface Activity of MWW Zeolite Nanosheets Prepared via a One-Step Synthesis. J. Am. Chem. Soc..

[5] Leonowicz M.E., Lawton J.A., Lawton S.L., Rubin M.K. (1994). MCM-22: A Molecular Sieve with Two Independent Multidimensional Channel Systems. Science.

[6] Roth W.J., Kresge C.T., Vartul J.C., Leonowicz M.E., Fung A.S., McCullen S.B. (1995). MCM-36: The first pillared molecular sieve with zeoliteproperties. Stud. Surf. Sci. Catal..

[7] Lawton S.L., Fung A.S., Kennedy G.J., Alemany L.B., Chang C.D., Hatzikos G.H., Lissy D.N., Rubin M.K., Timken H.-K.C., Steuernagel S. (1996). Zeolite MCM-49: A Three-Dimensional MCM-22 Analogue Synthesized byin SituCrystallization. J. Phys. Chem..

[8] Corma A., Diaz U., Fornés V., Guil J.M., Martínez-Triguero J., Creyghton E.J. (2000). Characterization and Catalytic Activity of MCM-22 and MCM-56 Compared with ITQ-2. J. Catal..

[9] Camblor M.A., Corma A., Diaz-Cabanas M.J., Baerlocher C. (1998). Synthesis and Structural Characterization of MWW Type Zeolite ITQ-1, the Pure Silica Analog of MCM-22 and SSZ-25. J. Phys. Chem. B.

[10] Corma A., Fornes V., Guil J.M., Pergher S., Maesen T.L.M., Buglass J.G. (2000). Preparation, characterisation and catalytic activity of ITQ-2, a delaminated zeolite. Microporous Mesoporous Mater..

[11] Zones S.I., Hwang S.-J., Davis M.E. (2001). Studies of the Synthesis of SSZ-25 Zeolite in a “Mixed-Template” System. Chemistry.

[12] Lin F., Ye Z., Kong L., Liu P., Zhang Y., Zhang H., Tang Y. (2022). Facile Morphology and Porosity Regulation of Zeolite ZSM-5 Mesocrystals with Synergistically Enhanced Catalytic Activity and Shape Selectivity. Nanomaterials.

[13] Zhang C., Lin F., Kong L., Ye Z., Pan D., Li H., Li H., Liu P., Zhang Y., Zhang H. (2022). c-Axis-penetrated mesoporous MWW zeolite nanosheets: Preparation by H_2_O_2_-induced micro-explosion and their enhanced properties. Inorg. Chem. Front..

[14] Li S., Yang H., Wang S., Wang J., Fan W., Dong M. (2022). Improvement of adsorption and catalytic properties of zeolites by precisely controlling their particle morphology. Chem. Commun..

[15] Hu W.-H., Liu M.-N., Luo Q.-X., Zhang J., Chen H., Xu L., Sun M., Ma X., Hao Q.-Q. (2023). Friedel-Crafts acylation of anisole with acetic anhydride over single- to multiple-layer MWW zeolites: Catalytic behavior and kinetic mechanism. Chem. Eng. J..

[16] Liang Y., Gao B., Zhou L., Yang X., Lu T., Yao H., Su Y. (2021). Rational construction of hierarchical SAPO-34 with enhanced MTO performance without an additional meso/macropore template. J. Mater. Chem. A.

[17] Dai W., Kouvatas C., Tai W., Wu G., Guan N., Li L., Valtchev V. (2021). Platelike MFI Crystals with Controlled Crystal Faces Aspect Ratio. J. Am. Chem. Soc..

[18] Ma Y., Hu J., Fan K., Chen W., Han S., Wu Q., Ma Y., Zheng A., Kunkes E., De Baerdemaeker T. (2023). Design of an Organic Template for Synthesizing ITR Zeolites under Ge-Free Conditions. J. Am. Chem. Soc..

[19] Ma Z., Deng H., Li L., Zhang Q., Chen G., Sun C., He H., Yu J. (2023). Fluoride-free and seed-free microwave-assisted hydrothermal synthesis of nanosized high-silica Beta zeolites for effective VOCs adsorption. Chem. Sci..

[20] Zhao L., Yang G., Hu H., Sun Y., Ma Z., Peng P., Ng E.-P., Tian P., Guo H., Svetlana M. (2022). SAPO-34 crystals with nanosheet morphology synthesized by pyrophosphoric acid as new phosphorus source. Microporous Mesoporous Mater..

[21] Asselman K., Kirschhock C., Breynaert E. (2023). Illuminating the Black Box: A Perspective on Zeolite Crystallization in Inorganic Media. Acc. Chem. Res..

[22] Ye Z., Zhao Y., Zhang H., Shi Z., Li H., Yang X., Wang L., Kong L., Zhang C., Sheng Z. (2022). Mesocrystal morphology regulation by “alkali metals ion switch”: Re-examining zeolite nonclassical crystallization in seed-induced process. J. Colloid Interface Sci..

[23] Li C., Liu Z., Goonetilleke E.C., Huang X. (2021). Temperature-dependent kinetic pathways of heterogeneous ice nucleation competing between classical and non-classical nucleation. Nat. Commun..

[24] Ivanova I.I., Andriako E.P., Kostyukov I.A., Zasukhin D.S., Fedosov D.A., Zarubin D.N. (2023). Multinuclear MAS NMR Monitoring of the Effect of Silicate Speciation on the Mechanism of Zeolite BEA Formation: Toward Engineering of Crystal Size and Morphology. Cryst. Growth Des..

[25] Jain R., Mallette A.J., Rimer J.D. (2021). Controlling Nucleation Pathways in Zeolite Crystallization: Seeding Conceptual Methodologies for Advanced Materials Design. J. Am. Chem. Soc..

[26] Wei P., Liu W., Li J., Wang Y., Yu Q., Yang Z., Liu X., Xu L., Li X., Zhu X. (2022). Synthesis and crystallization mechanism of EUO zeolite. Microporous Mesoporous Mater..

[27] Jain R., Niu Z., Choudhary M., Bourji H., Palmer J.C., Rimer J.D. (2023). In Situ Imaging of Faujasite Surface Growth Reveals Unique Pathways of Zeolite Crystallization. J. Am. Chem. Soc..

[28] Sheng Z., Li H., Du K., Gao L., Ju J., Zhang Y., Tang Y. (2021). Observing a Zeolite Nucleus (Subcrystal) with a Uniform Framework Structure and Its Oriented Attachment without Single-Molecule Addition. Angew. Chem. Int. Ed..

[29] Chawla A., Linares N., Li R., García-Martínez J., Rimer J.D. (2020). Tracking Zeolite Crystallization by Elemental Mapping. Chem. Mater..

[30] Zhang J., Bai R., Zhou Y., Chen Z., Zhang P., Li J., Yu J. (2022). Impact of a polymer modifier on directing the non-classical crystallization pathway of TS-1 zeolite: Accelerating nucleation and enriching active sites. Chem. Sci..

[31] Zhang X., Yang M., Wang L., Han J., Lou C., Xu S., Zhang Y., Wu R., Tian P., Liu Z. (2023). Recognizing the Minimum Structural Units Driving the Crystallization of SAPO-34 in a Top-Down Process. Chemistry.

[32] Bai R., Song Y., Latsch L., Zou Y., Feng Z., Coperet C., Corma A., Yu J. (2022). Switching between classical/nonclassical crystallization pathways of TS-1 zeolite: Implication on titanium distribution and catalysis. Chem. Sci..

[33] Choudhary M.K., Jain R., Rimer J.D. (2020). In situ imaging of two-dimensional surface growth reveals the prevalence and role of defects in zeolite crystallization. Proc. Natl. Acad. Sci. USA.

[34] Hong S., Mallette A.J., Neeway J.J., Motkuri R.K., Rimer J.D., Mpourmpakis G. (2023). Understanding formation thermodynamics of structurally diverse zeolite oligomers with first principles calculations. Dalton Trans..

[35] Hu G., Ma D., Liu L., Cheng M., Bao X. (2004). In situ assembly of zeolitic building blocks into high-order structures. Angew. Chem. Int. Ed..

[36] Liu H., Xu F., Gao Y., Wu P., Xu L. (2024). Construction of hierarchical Sn-ECNU-7 zeolite with MWW nanosheets for Baeyer-Villiger oxidation of bulky reactant. Microporous Mesoporous Mater..

[37] Xu S., Zhu M., Huang P., Zheng S., Qiao Y., Liang Y., Chen X., Kita H. (2024). Preparation and catalytic performance of the supported Ti-MWW zeolites. Microporous Mesoporous Mater..

[38] Zi W.W., Gao Z., Zhang J., Zhao B.X., Cai X.S., Du H.B., Chen F.J. (2020). An Extra-Large-Pore Pure Silica Zeolite with 16x8x8-Membered Ring Pore Channels Synthesized using an Aromatic Organic Directing Agent. Angew. Chem. Int. Ed..

[39] Zi W.W., Cai X.S., Jiao F., Du H.B. (2020). Synthesis, Structure and Properties of an Extra-Large-Pore Aluminosilicate Zeolite NUD-6. Chem. Eur. J..

