# Supplementary material for: Morphology Regulation of Zeolite MWW via Classical/Nonclassical Crystallization Pathways
Source: Molecules. 2023 Dec 27;29(1):170. doi: 10.3390/molecules29010170 (PMC10780621; doi:10.3390/molecules29010170)
Supplement: Supplementary file 1 [file molecules-29-00170-s001.zip › molecules-2774536-supplementary.pdf]

Morphology Regulation of Zeolite MWW via Classical/Nonclassical Crystallization Pathways

Wenwen Zi [1,*], Zejing Hu [1], Xiangyu Jiang [1], Junjun Zhang [2], Chengzhi Guo [1], Konggang Qu [1], Shuo Tao [1], Dengran Tan [1] and Fangling Liu [1]

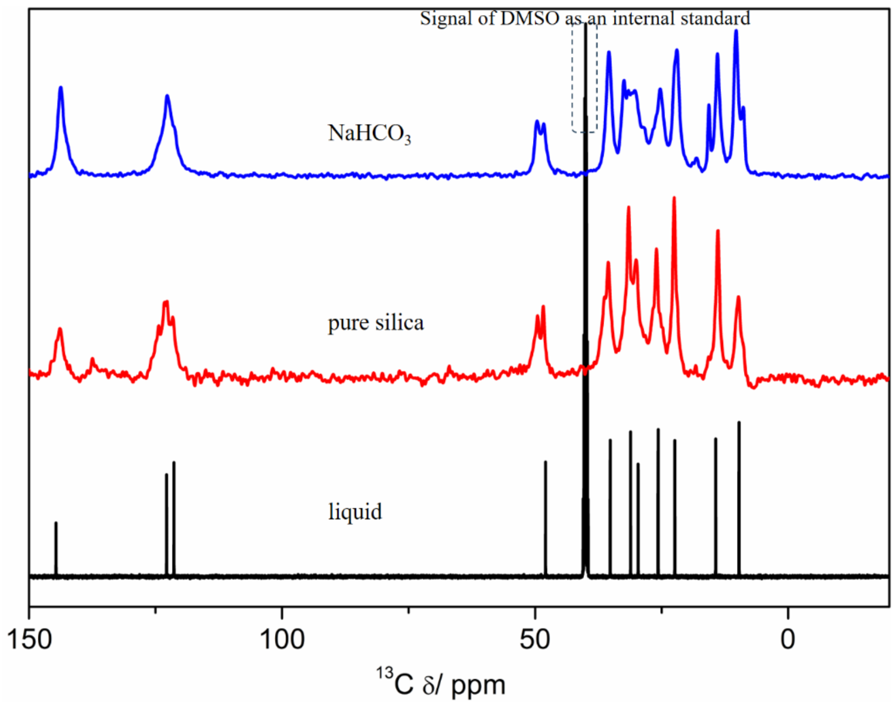

Figure S1. Comparison of ^{13}C NMR spectra of the synthesized sample and the liquid NMR in D_2O.

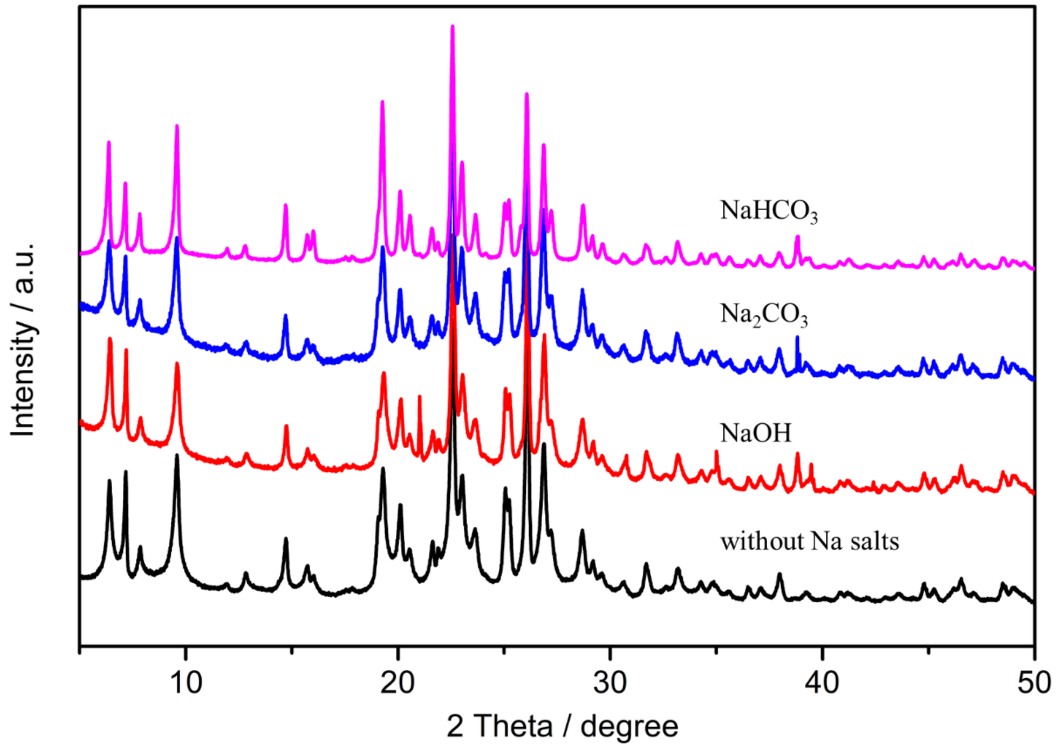

Figure S2. PXRD patterns of the samples with NaOH, Na_2CO_3, $NaHCO_3$.

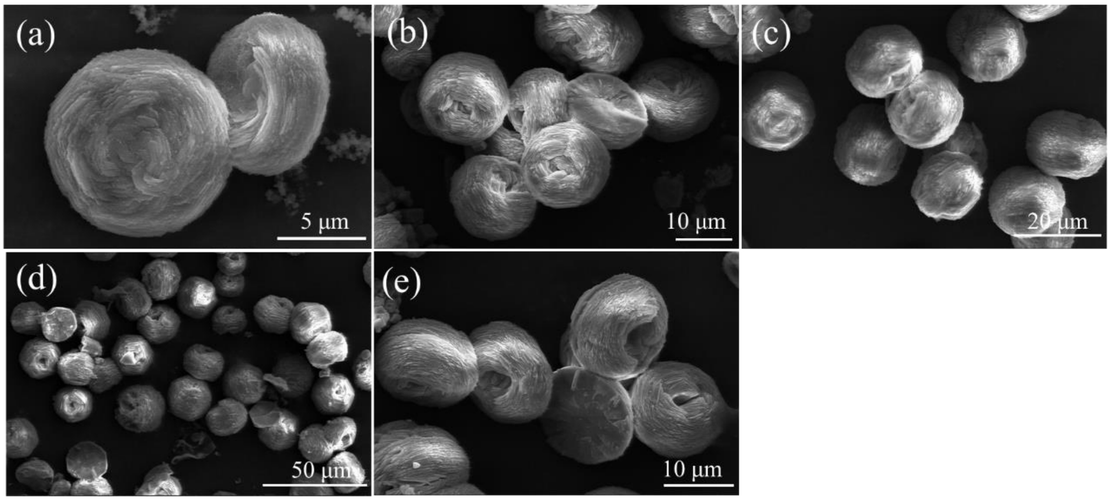

Figure S3. SEM images of the samples with different ratio of NaOH/Si (a: 0.05, b: 0.1, c: 0.15, d: 0.2, e: 0.25).

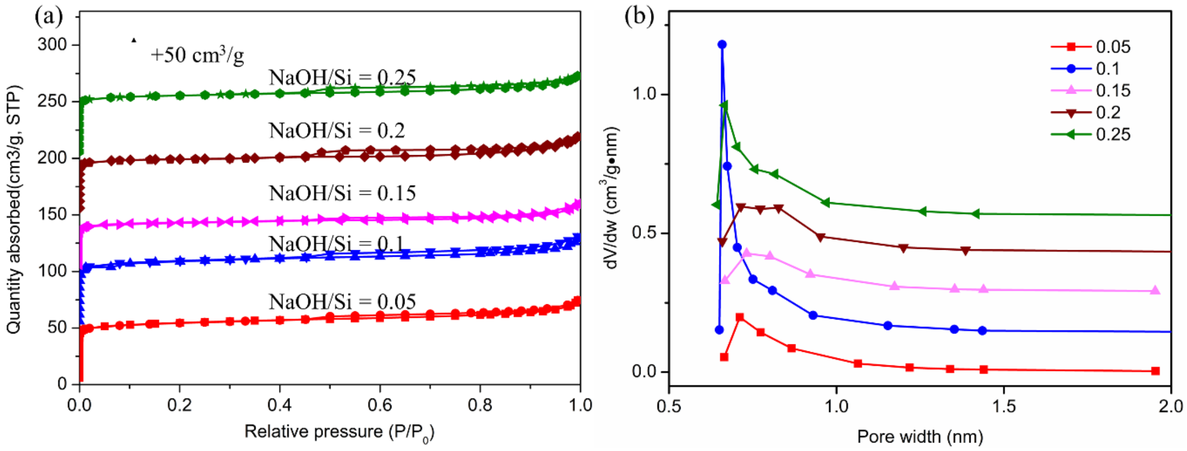

Figure S4. N_2 adsorption/desorption isotherms and pore size distribution of NaOH/Si.

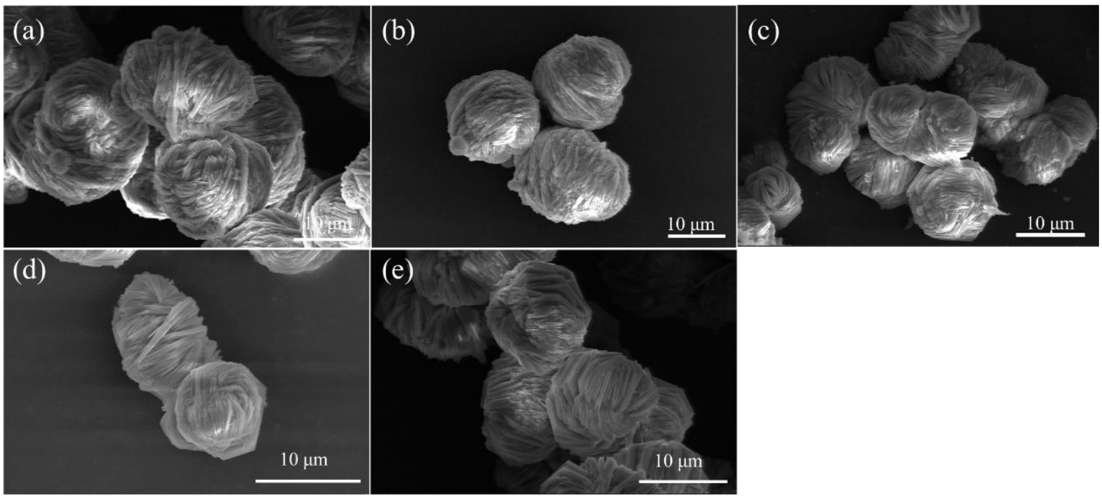

Figure S5. SEM images of the samples with different ratio of Na_2CO_3/Si (a: 0.05, b: 0.25, c: 0.1, d: 0.15, e: 0.2).

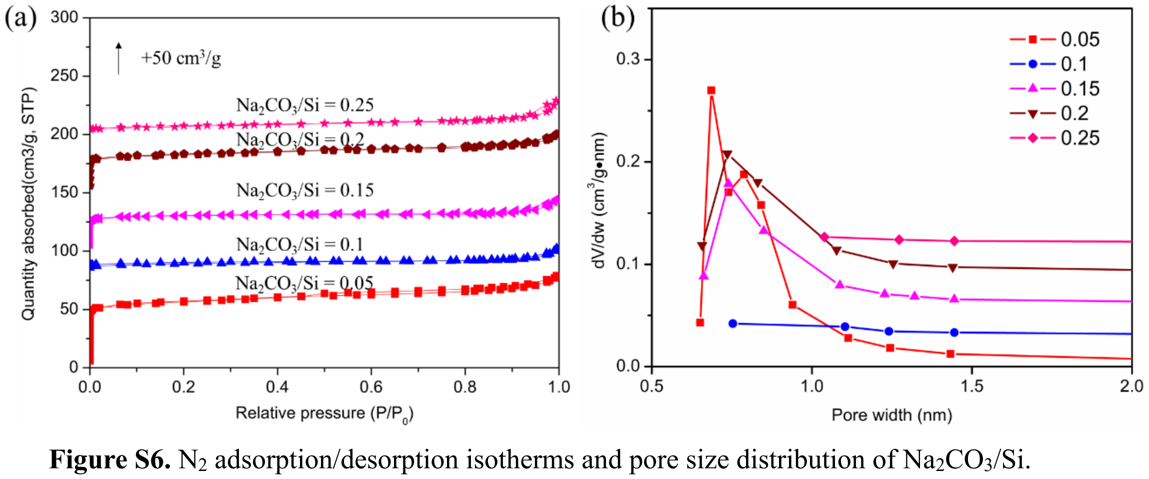

Figure S6. N_2 adsorption/desorption isotherms and pore size distribution of Na_2CO_3/Si.

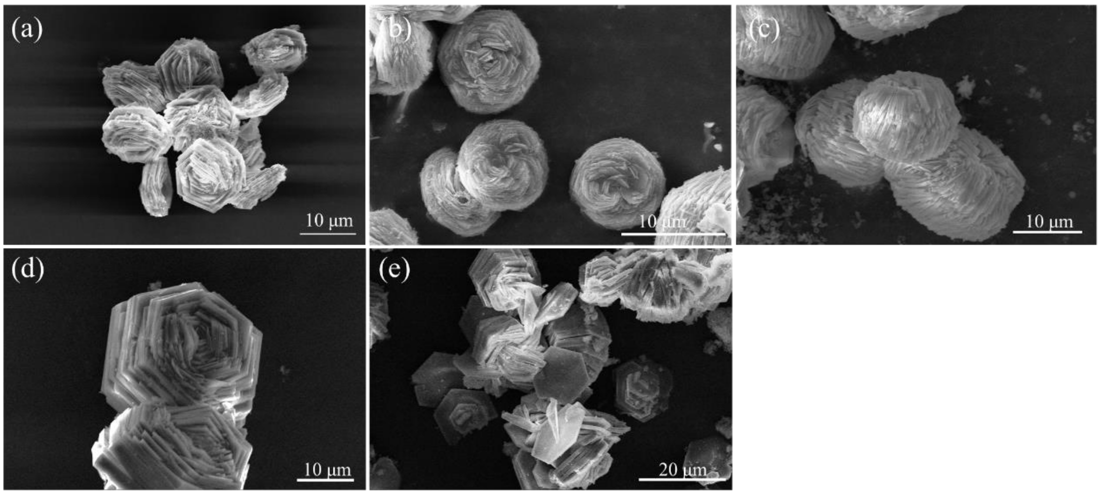

Figure S7. SEM images of the samples with the addition of $NaHCO_3$ under different ratio of HF/Si (a: 0.5, b: 0.65, c: 0.75, d: 1, e: 1.25).

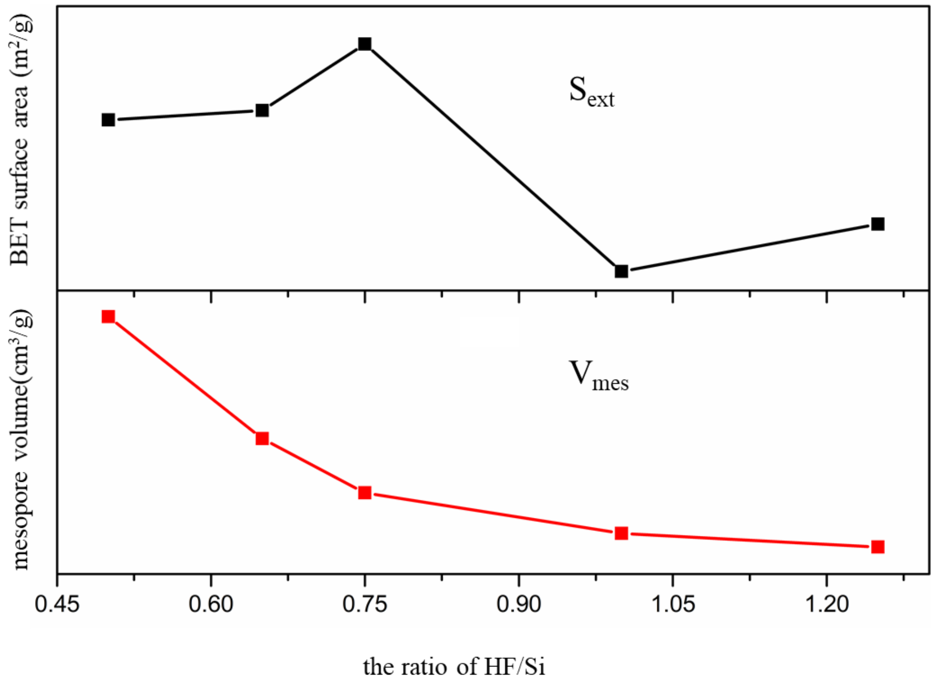

Figure S8. The effect of HF on the S_{ext} and V_{mes} in the $NaHCO_3$-MWW samples.

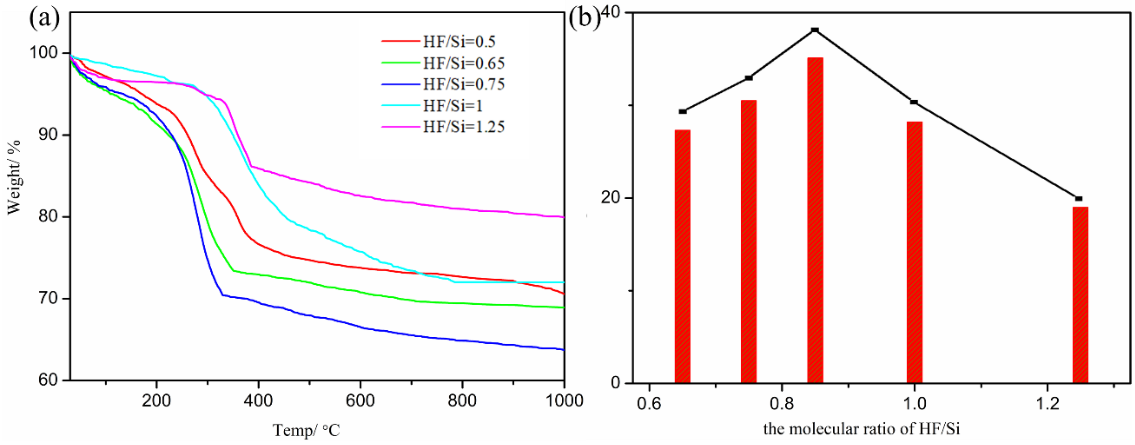

Figure S9. (a) TG analysis of the samples with the addition of $NaHCO_3$ under different ratio of HF/Si (a: 0.5, b: 0.65, c: 0.75, d: 1, e: 1.25) and (b) the mass loss comparation.

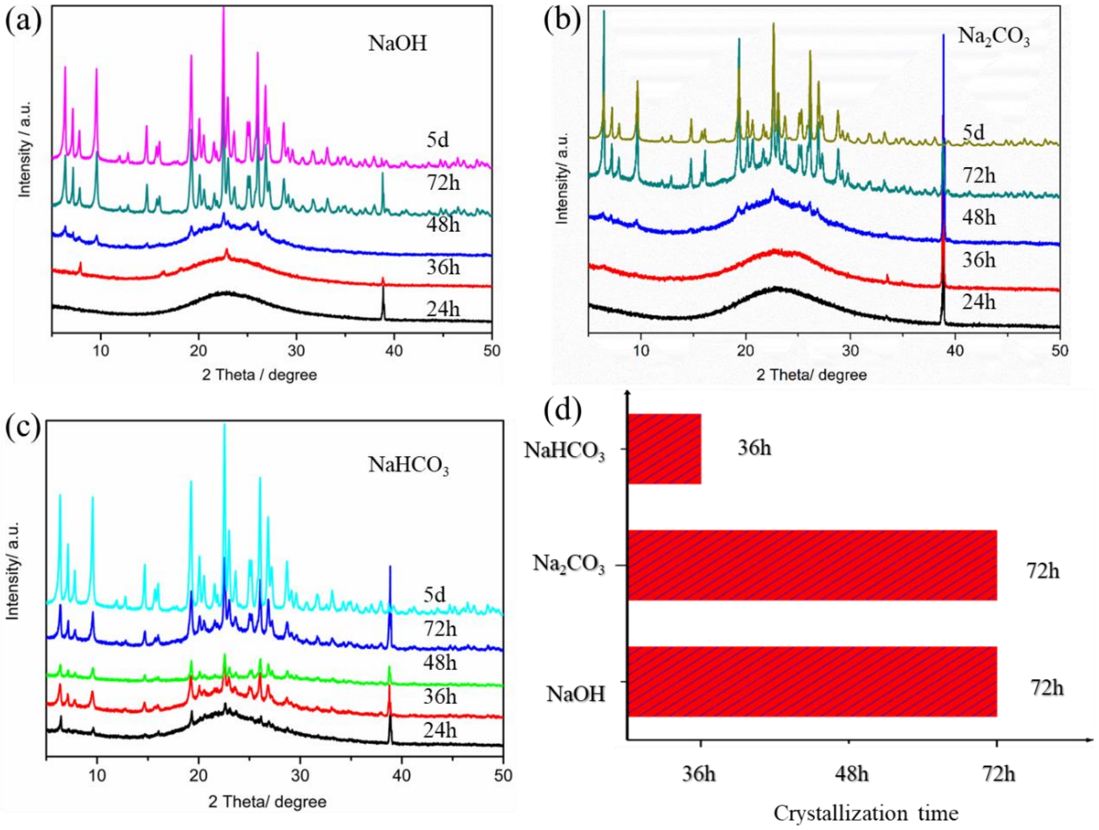

Figure S10. PXRD patterns of samples with different kinds of Na salts subjected to the crystallization time and the comparison of the nucleation time.

Table S1. EDS data for the samples with the addition of different ratio of NaOH/Si.

Run	NaOH/Si	Si(wt%)	O(wt%)	C(wt%)	N(wt%)	F(wt%)	Na(wt%)
1	0.05	50.17	30.46	16.32	2.02	0.67	0.36
2	0.1	39.21	40.95	15.21	3.02	1.31	0.30
3	0.15	49.84	30.15	19.66	1.33	0	0.02
4	0.2	48.90	34.74	13.71	2.36	0.29	0
5	0.25	41.00	41.77	13.81	2.72	0.62	0.08

Table S2. EDS data for the samples with the addition of different ratio of Na_2CO_3/Si.

Run	Na_2CO_3/Si	Si(wt%)	O(wt%)	C(wt%)	N(wt%)	F(wt%)	Na(wt%)
1	0.05	40.71	41.00	14.40	2.98	0.84	0.08
2	0.1	51.66	33.51	13.60	1.11	0	0.11
3	0.15	46.86	36.88	13.55	2.37	0.22	0.13
4	0.2	46.65	36.40	14.34	2.36	0.25	0
5	0.25	47.07	35.35	14.79	2.44	0.31	0.05

Table S3. EDS data for the samples with the addition of $NaHCO_3$ under different ratio of HF/Si.

Run	HF/Si	Si(wt%)	O(wt%)	C(wt%)	N(wt%)	F(wt%)	Na(wt%)
1	0.5	58.98	24.45	15.25	0.91	0.29	0.11
2	0.65	70.19	19.47	10.22	0	0.11	0.02
3	0.75	39.11	43.60	13.73	2.82	0.61	0.12
4	1	35.63	40.28	18.83	3.94	1.21	0.12
5	1.25	43.69	37.87	14.92	2.58	0.68	0.26
